# Association of preoperative and postoperative systemic inflammatory markers with early mortality and morbidity after CABG surgery

**DOI:** 10.1186/s13019-026-03928-x

**Published:** 2026-03-09

**Authors:** Rauf Önder, Salih Özçobanoğlu, Bengi Polen Gündoğdu, Veli Burak Taçkın, İsmail Kakillioğlu

**Affiliations:** 1 Yüksekova State Hospital, İpekyolu Street, İnanlı Region, Yüksekova/Hakkari, 30300 Turkey; 2 Akdeniz University, Konyaaltı, Antalya, Turkey; 3 Antalya City Hospital, Kepez, Antalya, Turkey

**Keywords:** Coronary artery bypass grafting, Neutrophil-to-lymphocyte ratio, Platelet-to-lymphocyte ratio, Systemic immune-inflammation index, Inflammation, Early outcomes, Mortality

## Abstract

**Objective:**

This study aimed to investigate the relationship between preoperative and postoperative inflammatory parameters—including neutrophil-to-lymphocyte ratio (NLR), platelet-to-lymphocyte ratio (PLR), and systemic immune-inflammation index (SII)—and early clinical outcomes in patients undergoing isolated coronary artery bypass graft (CABG) surgery.

**Methods:**

In this single-center, retrospective clinical study, data from 500 adult patients who underwent elective, isolated on-pump CABG surgery between 2018 and 2023 were analyzed. Preoperative and postoperative (day 1 and day 7) complete blood count parameters, as well as early postoperative clinical outcomes (including mortality, atrial fibrillation, length of ICU and hospital stay, and other complications), were systematically evaluated. Multivariate logistic regression was used to identify independent predictors of early mortality.

**Results:**

Patients who experienced early mortality had significantly higher preoperative NLR, PLR, SII, blood urea nitrogen, creatinine, and older age, as well as longer cardiopulmonary bypass and cross-clamp times. Postoperative day 7 values for NLR, PLR, SII, and neutrophil count were also significantly elevated in the mortality group, while lymphocyte and platelet counts were lower. Both preoperative and postoperative inflammatory markers were positively correlated with prolonged extubation time, length of ICU and hospital stay, and the occurrence of atrial fibrillation. In multivariate analysis, preoperative NLR, PLR, SII, and postoperative day 7 NLR and SII emerged as independent predictors of early mortality.

**Conclusion:**

Elevated preoperative and postoperative inflammatory markers (NLR, PLR, and SII) are significantly associated with adverse early clinical outcomes after CABG surgery. Monitoring these parameters may facilitate early risk stratification and improve postoperative patient management.

## Introduction

Coronary artery disease is one of the leading causes of morbidity and mortality worldwide, and surgical revascularization techniques are widely used in its treatment [1]. Coronary artery bypass graft (CABG) surgery is frequently preferred, particularly in patients with multivessel disease, with the aim of reducing myocardial ischemia and improving quality of life [2]. Early prediction of complications that may arise in the postoperative period in these patients is of great importance both for patient management and for the efficiency of healthcare services.

In recent years, certain hematological parameters used to evaluate systemic inflammatory response have been shown to be associated with complications that may occur following surgical interventions [3, 4]. The neutrophil-to-lymphocyte ratio (NLR), platelet-to-lymphocyte ratio (PLR), and systemic immune-inflammation index (SII), which can be easily obtained from a complete blood count, are considered reliable indicators reflecting the degree of inflammation [5]. It is suggested that preoperative levels of these parameters may be associated with adverse events that may develop postoperatively [6].

Cardiopulmonary bypass, reperfusion injury, systemic inflammatory response, and hemodynamic changes are pathophysiological processes specific to CABG surgery that may lead to serious early postoperative complications such as organ dysfunction, arrhythmias, infections, and mortality [7, 8]. Therefore, the use of biomarkers that provide insight into the patient’s inflammatory status in the preoperative period may contribute significantly to risk stratification and patient management.

This study aimed to investigate the relationship between preoperative and postoperative inflammatory parameters—including neutrophil-to-lymphocyte ratio (NLR), platelet-to-lymphocyte ratio (PLR), and systemic immune-inflammation index (SII)—and early clinical outcomes in patients undergoing isolated coronary artery bypass graft (CABG) surgery.

## Materials and methods

### Study design and patient population

This single-center, retrospective clinical study was conducted at the Department of Cardiovascular Surgery, Akdeniz University Faculty of Medicine. The study included patients who underwent coronary artery bypass grafting (CABG) surgery between 2018 and 2023. Ethical approval was obtained from the Akdeniz University Clinical Research Ethics Committee (KAEK-858, 15.11.2023). The requirement for informed consent was waived due to the retrospective nature of the study. Data from a total of 500 patients were evaluated based on information extracted from the electronic medical record system (MIA-MED), operative notes, and discharge summaries.

### Inclusion and exclusion criteria

**Fig. 1:**
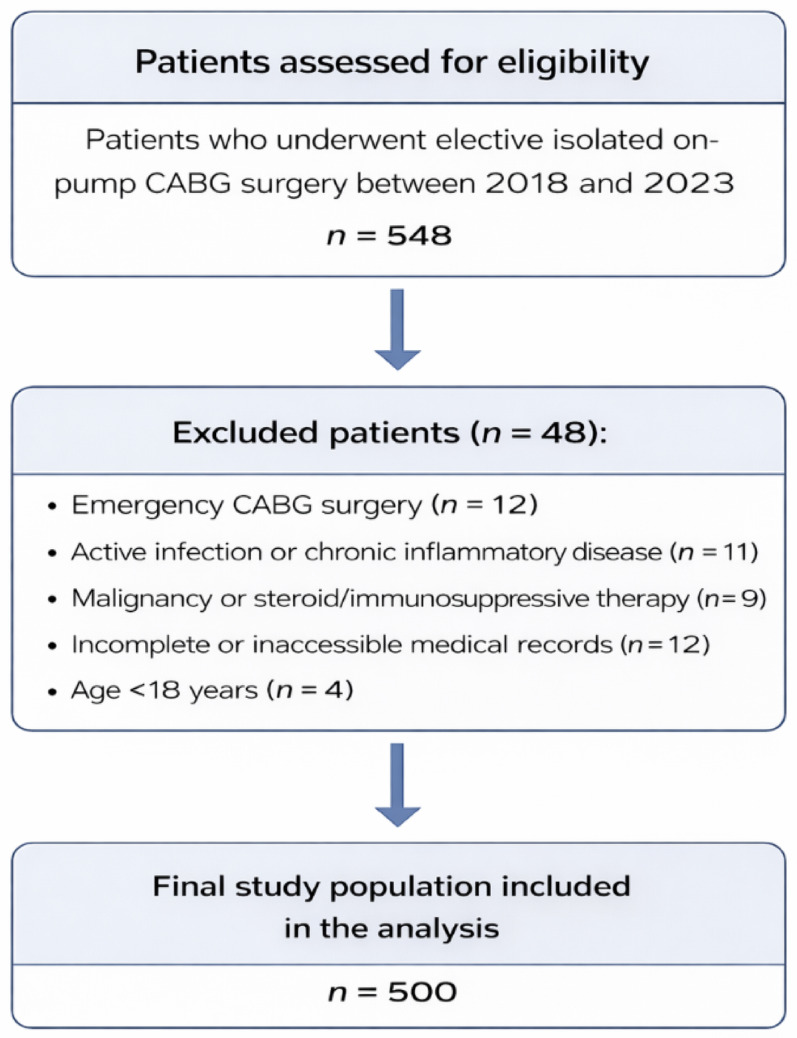
Flow diagram of patient selection and study population

A total of 548 patients who underwent isolated, elective on-pump coronary artery bypass grafting (CABG) between 2018 and 2023 were initially assessed for eligibility. The inclusion criteria were adult patients (≥ 18 years) with complete preoperative complete blood count data available for analysis. Patients were excluded if they underwent concomitant cardiac surgical procedures or emergency CABG surgery, or had active infection, hematological disorders, chronic inflammatory diseases, or malignancy. Additional exclusion criteria included the use of steroid or immunosuppressive therapy, age under 18 years, and incomplete or inaccessible medical records. After applying these criteria, a total of 500 patients were included in the final analysis (Fig. 1).

### Anesthesia management

All patients underwent general anesthesia following standard preoperative monitoring, including ECG, SpO₂, and invasive arterial blood pressure measurement. Anesthesia induction was achieved with intravenous midazolam, etomidate, and fentanyl; muscle relaxation was provided using either rocuronium or pancuronium. After endotracheal intubation, controlled mechanical ventilation was maintained, with respiratory parameters meticulously adjusted according to each patient’s physiological status.

Anesthesia was maintained with volatile agents such as isoflurane or sevoflurane and short-acting opioids like remifentanil. Intraoperative fluid management, electrolyte balance, and arterial blood gas analyses were regularly assessed to ensure hemodynamic stability. Prior to initiation of cardiopulmonary bypass, systemic anticoagulation was achieved with intravenous heparin, targeting an activated clotting time (ACT) greater than 480 s. Upon completion of the surgical procedure, the effect of heparin was reversed with protamine sulfate.

### Surgical procedure

All surgical procedures were performed via median sternotomy under cardiopulmonary bypass (CPB). Arterial cannulation was typically established in the ascending aorta, while venous cannulation was performed in the right atrium. During CPB, non-pulsatile flow was utilized, and systemic hypothermia was maintained at approximately 32 °C. Cardiac arrest was achieved using cold crystalloid antegrade cardioplegia; retrograde cardioplegia was also administered intraoperatively as needed. The targeted distal coronary anastomoses were performed first, followed by the proximal anastomoses. The number of distal anastomoses varied according to the patient’s coronary anatomy. Graft materials included the internal mammary artery, saphenous vein, and radial artery. After completion of all anastomoses, the aortic cross-clamp was removed and the heart was reperfused. Defibrillation was performed when necessary, and after achieving stable hemodynamic parameters, CPB was terminated. Following sternal closure, all patients were transferred to the intensive care unit, where postoperative management was conducted according to standardized protocols.

### Data collection and evaluation

Demographic data such as age and sex, as well as medical histories including hypertension, diabetes mellitus, dyslipidemia, COPD, chronic kidney disease, and previous cardiac surgery were recorded for all patients. Intraoperative and postoperative data related to the surgical procedure—such as the number of distal anastomoses, cross-clamp time, CPB duration, distinction between off-pump/on-pump procedures (all patients were on-pump), ICU and hospital length of stay, and duration of intubation—were also evaluated. Laboratory parameters recorded from the complete blood count included hemoglobin, hematocrit, leukocyte, platelet, lymphocyte, neutrophil, CRP, BUN, creatinine, AST, ALT, sodium, and potassium levels. These values were analyzed at three different time points: preoperatively, and on postoperative days 1 and 7. Ratios such as NLR (neutrophil/lymphocyte), PLR (platelet/lymphocyte), and SII ((neutrophil × platelet)/lymphocyte) were calculated based on these data. In addition, early postoperative clinical outcomes—including atrial fibrillation, myocardial infarction, low cardiac output syndrome, need for reoperation, infection, pulmonary complications, renal failure, thromboembolic events, cerebrovascular events, and mortality—were systematically assessed. Preoperative blood samples were obtained within 24 h before surgery. Postoperative day 1 samples were collected approximately 18–30 h after surgery, while postoperative day 7 samples were obtained within the postoperative day 7, according to routine clinical workflow.

### Statistical analysis

All statistical analyses were performed using IBM SPSS Statistics version 27.0 for Windows (IBM Corp., Armonk, NY, USA). The normality of the distribution of continuous variables was assessed using the Kolmogorov–Smirnov test. Continuous variables were expressed as mean ± standard deviation (SD), while categorical variables were presented as numbers and percentages. For the comparison of continuous variables between groups, the independent samples t-test was used for normally distributed variables and the Mann–Whitney U test for non-normally distributed variables. Categorical variables were compared using the Chi-square test or Fisher’s exact test where appropriate. Correlation analyses between preoperative inflammatory parameters and clinical outcomes were performed using Pearson or Spearman correlation coefficients, based on the distribution of the data. Receiver operating characteristic (ROC) curve analysis was conducted to evaluate the predictive accuracy of inflammatory markers and perioperative variables for in-hospital mortality. The area under the curve (AUC), optimal cut-off values (calculated using Youden’s index), sensitivity, and specificity were reported. Variables found to be statistically significant in univariate analyses were included in a stepwise multivariate logistic regression model to identify independent predictors of mortality. The results of the regression analysis were presented as adjusted odds ratios (OR) with 95% confidence intervals (CI). Statistical significance was set at *p* < 0.05.

## Results

Patients who experienced in-hospital mortality were significantly older (66.1 ± 7.2 vs. 61.3 ± 8.8 years, *p* = 0.013) and had longer cardiopulmonary bypass time (182.9 ± 79.0 vs. 125.7 ± 45.1 min, *p* < 0.001) and cross-clamp time (106.7 ± 25.1 vs. 76.5 ± 24.7 min, *p* < 0.001). ICU stay [5.0 (4.0–6.0) vs. 4.0 (3.0–5.0) days, *p* = 0.003], extubation time [6.0 (6.0–8.0) vs. 5.0 (4.0–6.0) h, *p* < 0.001], and hospital stay [10.0 (8.0–15.0) vs. 8.0 (7.0–10.0) days, *p* < 0.001] were also significantly longer in the mortality group. Among preoperative laboratory parameters, BUN (23.6 ± 11.5 vs. 18.2 ± 9.0 mg/dL, *p* = 0.015) and creatinine (1.5 ± 1.1 vs. 1.1 ± 1.0 mg/dL, *p* = 0.017) levels were significantly higher in patients with mortality. Preoperative inflammatory indices were also significantly elevated, including NLR [3.1 (2.5–4.0) vs. 2.2 (1.7–3.0), *p* = 0.001], PLR [148.0 (119.3–189.9) vs. 111.2 (87.4–146.2), *p* = 0.002], and SII (×10⁴) [83.7 (45.4–93.8) vs. 53.4 (37.6–78.9), *p* = 0.040]. Conversely, preoperative lymphocyte count was significantly lower in the mortality group (*p* < 0.001). Other demographic, echocardiographic, and biochemical parameters did not differ significantly between the groups (Table 1).

**Table 1:** The Relationship Between Preoperative Demographic, Clinical, and Hematological Parameters and Mortality

	Mortality (-)	Mortality (+)	
	Mean ± S.D.	Mean ± S.D.	*p* value
**Age (years)**	61.3 ± 8.8	66.1 ± 7.2	**0.013**
**CPB time (min)**	125.7 ± 45.1	182.9 ± 79.0	**< 0.001**
**Cross-clamp time (min)**	76.5 ± 24.7	106.7 ± 25.1	**< 0.001**
**ICU stay (days)**	4.0 (3.0–5.0)	5.0 (4.0–6.0)	**0.003**
**Extubation time (h)**	5.0 (4.0–6.0)	6.0 (6.0–8.0)	**< 0.001**
**Hospital stay (days)**	8.0 (7.0–10.0)	10.0 (8.0–15.0)	**< 0.001**
**EF (%)**	59.4 ± 8.3	55.8 ± 7.4	**0.004**
**LA (mm)**	37.6 ± 4.8	39.4 ± 5.6	0.235
**LVESD (mm)**	48.3 ± 5.3	48.0 ± 4.5	0.968
**LVEDD (mm)**	32.0 ± 6.0	30.4 ± 5.4	0.127
**Pre-op Hb (g/dL)**	13.3 ± 1.8	12.8 ± 1.9	0.242
**Pre-op HCT (%)**	41.5 ± 2.5	38.4 ± 5.3	0.314
**Pre-op WBC (10³/µL)**	8.6 ± 3.2	8.3 ± 1.8	0.861
**Pre-op PLT (10³/µL)**	258.5 ± 134.1	242.4 ± 77.6	0.683
**Pre-op Lymphocyte (10³/µL)**	2.3 ± 0.8	1.5 ± 0.2	**< 0.001**
**Pre-op Neutrophil (10³/µL)**	5.3 ± 2.8	5.1 ± 1.6	0.970
**Pre-op CRP (mg/L)**	14.2 ± 36.0	12.9 ± 18.4	0.435
**Pre-op BUN (mg/dL)**	18.2 ± 9.0	23.6 ± 11.5	**0.015**
**Pre-op Creatinine (mg/dL)**	1.1 ± 1.0	1.5 ± 1.1	**0.017**
**Pre-op Sodium (mmol/L)**	136.3 ± 8.5	136.6 ± 3.8	0.623
**Pre-op Potassium (mmol/L)**	4.5 ± 2.5	4.5 ± 0.6	0.239
**Pre-op AST (U/L)**	26.2 ± 35.4	34.4 ± 34.2	0.191
**Pre-op ALT (U/L)**	25.0 ± 18.6	40.5 ± 70.6	0.997
**Pre-op NLR**	2.2 (1.7–3.0)	3.1 (2.5–4.0)	**0.001**
**Pre-op PLR**	111.2 (87.4–146.2)	148.0 (119.3–189.9)	**0.002**
**Pre-op SII(×10⁴)**	53.4 (37.6–78.9)	83.7 (45.4–93.8)	**0.040**

On postoperative day 7, patients in the mortality group had significantly lower hemoglobin (9.7 ± 1.4 vs. 10.7 ± 1.2 g/dL, *p* = 0.006) and platelet counts (238.3 ± 59.6 vs. 294.6 ± 96.6 10³/µL, *p* = 0.007). In contrast, neutrophil count (11.9 ± 6.4 vs. 7.4 ± 3.2 10³/µL, *p* = 0.003), BUN (59.4 ± 31.5 vs. 22.1 ± 13.4 mg/dL, *p* < 0.001), creatinine (2.6 ± 1.7 vs. 1.1 ± 0.7 mg/dL, *p* < 0.001), sodium (143.0 ± 10.4 vs. 134.7 ± 3.6 mmol/L, *p* = 0.005), and AST (326.3 ± 643.3 vs. 31.1 ± 45.0 U/L, *p* = 0.004) levels were significantly higher in the mortality group. Postoperative inflammatory indices on day 7 were also markedly elevated in patients with mortality, including NLR [8.8 (6.1–14.5) vs. 3.3 (2.4–4.5), *p* < 0.001], PLR [288.2 (139.3–359.8) vs. 140.0 (107.6–183.8), *p* = 0.002], and SII (×10⁴) [168.0 (122.6–374.5) vs. 92.8 (66.3–135.9), *p* < 0.001]. Additionally, lymphocyte count was significantly lower in the mortality group (1.2 ± 0.8 vs. 2.1 ± 0.9 10³/µL, *p* < 0.001) (Table 2).

**Table 2:** The Relationship Between Postoperative Day 1 and Day 7 Laboratory Parameters and Mortality

	Mortality (-)	Mortality (+)	
	Mean ± S.D.	Mean ± S.D.	*p* value
**Post-op 1. Day Hb (g/dL)**	10.3 ± 1.2	9.7 ± 1.7	0.113
**Post-op 1. Day HCT (%)**	30.2 ± 3.6	28.4 ± 5.0	0.140
**Post-op 1. Day WBC (10³/µL)**	14.2 ± 4.8	15.4 ± 4.1	0.182
**Post-op 1. Day PLT (10³/µL)**	255.8 ± 75.3	313.3 ± 36.2	0.767
**Post-op 1. Day Lymphocyte (10³/µL)**	0.7 ± 0.6	1.3 ± 0.2	0.076
**Post-op 1. Day Neutrophil (10³/µL)**	12.4 ± 4.3	13.3 ± 3.9	0.242
**Post-op 1. Day CRP (mg/L)**	26.3 ± 41.8	13.1 ± 9.9	**0.048**
**Post-op 1. Day BUN (mg/dL)**	19.7 ± 8.6	27.7 ± 15.0	**0.002**
**Post-op 1. Day Creatinine (mg/dL)**	1.3 ± 3.6	1.8 ± 1.3	**< 0.001**
**Post-op 1. Day Sodium (mmol/L)**	140.2 ± 3.4	142.8 ± 5.4	**0.027**
**Post-op 1. Day Potassium (mmol/L)**	4.3 ± 0.5	4.5 ± 0.8	0.096
**Post-op 1. Day AST (U/L)**	58.2 ± 34.1	135.9 ± 186.0	0.192
**Post-op 1. Day ALT (U/L)**	40.4 ± 26.6	44.8 ± 39.1	0.701
**Post-op 1. Day NLR**	20.0 (12.0–27.8)	15.9 (11.1–27.6)	0.367
**Post-op 1. Day PLR**	409.9 (251.0–613.0)	328.9 (170.0–547.6)	0.122
**Post-op 1. Day SII (×10⁴)**	486.4 (273.5–722.6)	389.3 (294.3–704.8)	0.376
**Post-op 7. Day Hb (g/dL)**	10.7 ± 1.2	9.7 ± 1.4	**0.006**
**Post-op 7. Day HCT (%)**	32.0 ± 3.5	30.4 ± 4.3	0.090
**Post-op 7. Day WBC (10³/µL)**	11.8 ± 17.4	13.9 ± 7.2	0.126
**Post-op 7. Day PLT (10³/µL)**	294.6 ± 96.6	238.3 ± 59.6	**0.007**
**Post-op 7. Day Lymphocyte (10³/µL)**	2.1 ± 0.9	1.2 ± 0.8	**< 0.001**
**Post-op 7. Day Neutrophil (10³/µL)**	7.4 ± 3.2	11.9 ± 6.4	**0.003**
**Post-op 7. Day CRP (mg/L)**	77.5 ± 52.1	99.8 ± 88.0	0.623
**Post-op 7. Day BUN (mg/dL)**	22.1 ± 13.4	59.4 ± 31.5	**< 0.001**
**Post-op 7. Day Creatinine (mg/dL)**	1.1 ± 0.7	2.6 ± 1.7	**< 0.001**
**Post-op 7. Day Sodium (mmol/L)**	134.7 ± 3.6	143.0 ± 10.4	**0.005**
**Post-op 7. Day Potassium (mmol/L)**	4.1 ± 0.5	4.2 ± 1.3	0.537
**Post-op 7. Day AST (U/L)**	31.1 ± 45.0	326.3 ± 643.3	**0.004**
**Post-op 7. Day ALT (U/L)**	43.8 ± 58.7	327.2 ± 621.2	0.639
**Post-op 7. Day NLR**	3.3 (2.4–4.5)	8.8 (6.1–14.5)	**< 0.001**
**Post-op 7. Day PLR**	140.0 (107.6–183.8)	288.2 (139.3–359.8)	**0.002**
**Post-op 7. Day SII (×10⁴)**	92.8 (66.3–135.9)	168.0 (122.6–374.5)	**< 0.001**

Patients who developed postoperative atrial fibrillation had significantly higher preoperative inflammatory indices, including NLR [3.6 (3.1–4.7) vs. 2.0 (1.6–2.5), *p* < 0.001], PLR [156.4 (122.8–202.9) vs. 105.7 (82.5–133.5), *p* < 0.001], and SII (×10⁴) [94.6 (70.3–139.7) vs. 47.9 (35.2–65.4), *p* < 0.001]. Postoperative inflammatory markers were also elevated in the AF group on day 1 and day 7, including post-op day 1 NLR [23.2 (14.7–33.8) vs. 18.9 (11.7–26.9), *p* = 0.002] and SII (×10⁴) [600.3 (372.1–895.6) vs. 456.2 (266.5–704.2), *p* < 0.001], as well as post-op day 7 NLR [4.3 (3.2–6.1) vs. 3.2 (2.3–4.3), *p* < 0.001], PLR [162.2 (120.0–229.0) vs. 134.0 (103.7–176.7), *p* < 0.001], and SII (×10⁴) [121.4 (80.7–163.7) vs. 89.7 (61.3–128.0), *p* < 0.001]. In addition, ICU stay was significantly longer in patients with AF [5.0 (5.0–6.0) vs. 3.0 (3.0–4.0) days, *p* < 0.001] (Table 3).

**Table 3:** The Relationship Between Atrial Fibrillation and Inflammatory Parameters

	Atrial Fibrillation (-)	Atrial Fibrillation (+)	
	Mean ± S.D.	Mean ± S.D.	*p* value
**Cross-clamp time (min)**	77.1 ± 24.8	79.8 ± 27.7	0.212
**ICU stay (days)**	3.0 (3.0–4.0)	5.0 (5.0–6.0)	**< 0.001**
**Pre-op NLR**	2.0 (1.6–2.5)	3.6 (3.1–4.7)	**< 0.001**
**Pre-op PLR**	105.7 (82.5–133.5)	156.4 (122.8–202.9)	**< 0.001**
**Pre-op SII**	47.9 (35.2–65.4)	94.6 (70.3–139.7)	**< 0.001**
**Post-op 1. Day NLR**	18.9 (11.7–26.9)	23.2 (14.7–33.8)	**0.002**
**Post-op 1. Day PLR**	398.4 (246.3–590.6)	487.8 (251.0–667.5)	0.084
**Post-op 1. Day SII**	456.2 (266.5–704.2)	600.3 (372.1–895.6)	**< 0.001**
**Post-op 7. Day NLR**	3.2 (2.3–4.3)	4.3 (3.2–6.1)	**< 0.001**
**Post-op 7. Day PLR**	134.0 (103.7–176.7)	162.2 (120.0–229.0)	**< 0.001**
**Post-op 7. Day SII**	89.7 (61.3–128.0)	121.4 (80.7–163.7)	**< 0.001**

**Fig. 2:**
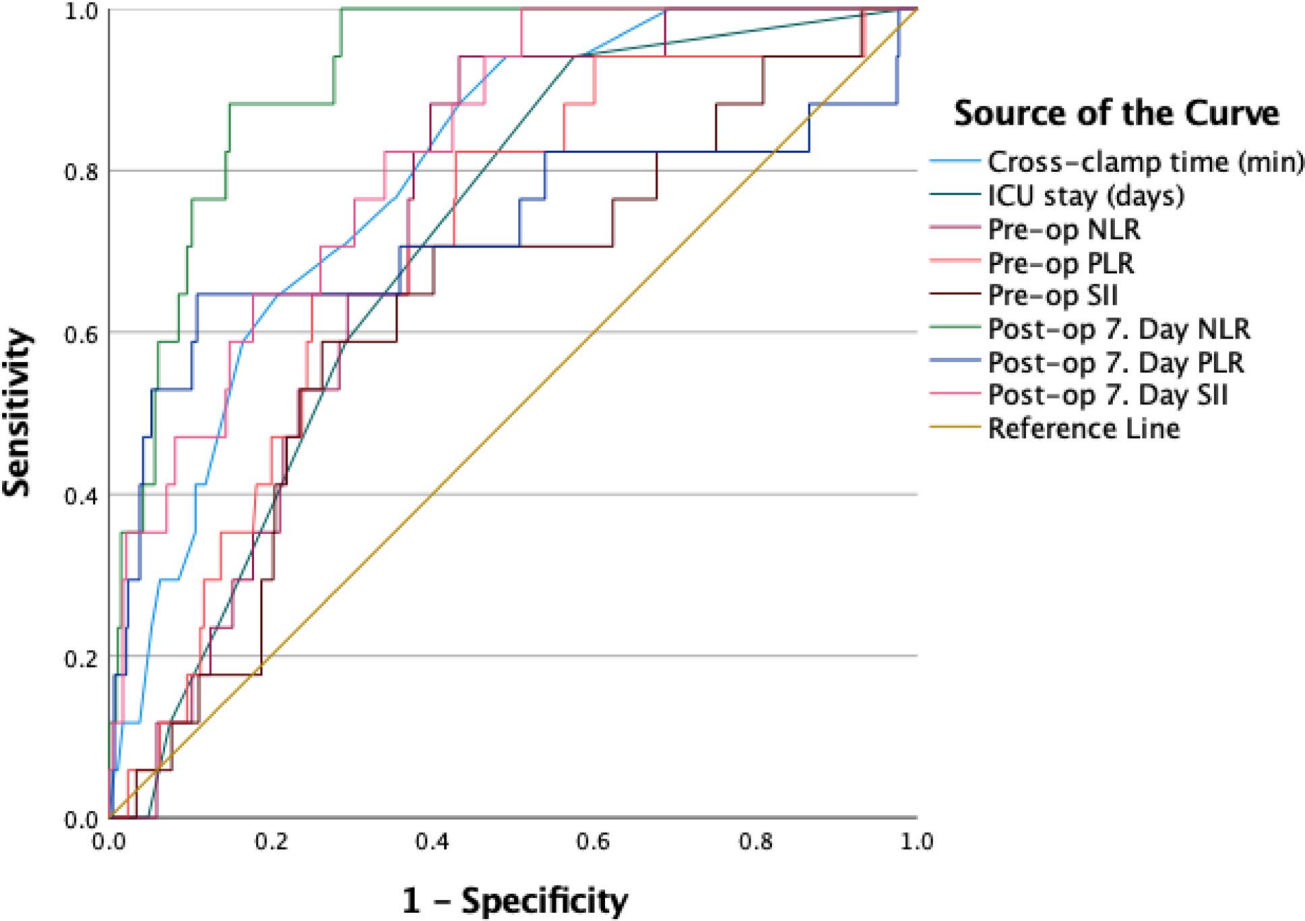
Comparison of ROC Curve Analysis Results

Postoperative day 7 NLR and SII curves demonstrate the highest accuracy and stand out among the predictive parameters for mortality (Fig. 2).

Postoperative day 7 NLR (AUC = 0.917, cut-off = 5.35, 88% sensitivity, 87% specificity, *p* < 0.001) and postoperative day 7 SII (AUC = 0.823, cut-off = 1577.4, 65% sensitivity, 83% specificity, *p* < 0.001) demonstrated the highest accuracy for predicting mortality. Cross-clamp time (AUC = 0.798), preoperative NLR (AUC = 0.732), and ICU stay (AUC = 0.706) also showed strong predictive value (Table-4).

**Table 4:** ROC Analysis Results for Predicting Mortality

	AUC	Cut-off	Sensitivity	Specificity	*p* value	Asymptotic 95% CI	
						Lower Bound	Upper Bound
**Cross-clamp time (min)**	0.798	86.5	%71	%73	**< 0.001**	0.711	0.885
**ICU stay (days)**	0.706	4.5	%60	%70	**0.004**	0.609	0.803
**Pre-op NLR**	0.732	2.54	%83	%62	**0.001**	0.652	0.813
**Pre-op PLR**	0.706	127.6	%70	%61	**0.004**	0.594	0.819
**Pre-op SII**	0.631	610.3	%65	%60	**0.047**	0.499	0.763
**Post-op 7. Day NLR**	0.917	5.35	%88	%87	**< 0.001**	0.873	0.961
**Post-op 7. Day PLR**	0.725	162.3	%71	%64	**0.002**	0.558	0.893
**Post-op 7. Day SII**	0.823	1577.4	%65	%83	**< 0.001**	0.740	0.907

Preoperative NLR, PLR, and SII values were positively and significantly correlated with extubation time, hospital stay, and ICU stay (all *p* < 0.001). Preoperative NLR demonstrated the strongest correlation with extubation time (*r* = 0.525) (Table-5).

**Table 5:** Correlation of Preoperative Inflammatory Parameters with Clinical Outcomes

		Extubation time (h)	Hospital stay (days)	ICU stay (days)
**Pre-op NLR**	Correlation	0.525	0.434	0.428
	p value	**< 0.001**	**< 0.001**	**< 0.001**
**Pre-op PLR**	Correlation	0.401	0.426	0.186
	p value	**< 0.001**	**< 0.001**	**< 0.001**
**Pre-op SII**	Correlation	0.479	0.470	0.362
	p value	**< 0.001**	**< 0.001**	**< 0.001**

In the stepwise multivariate logistic regression analysis, ICU stay (OR = 1.483, 95% CI: 1.184–1.858, *p* < 0.001), hospital stay (OR = 1.218, *p* = 0.021), preoperative BUN (OR = 1.135, *p* = 0.034), pre-op NLR (OR = 1.341, *p* = 0.004), pre-op PLR (OR = 1.127, *p* = 0.029), logSII (pre-op, *p* = 0.015), postoperative day 7 creatinine (OR = 4.755, *p* < 0.001), postoperative day 7 NLR (OR = 1.219, *p* = 0.006), and logSII (post-op day 7, *p* = 0.042) were identified as independent predictors of mortality (Table-6).

**Table 6:** Stepwise Multivariate Logistic Regression Model for Independent Predictors of Mortality

ICU stay (days)	Adjusted OR	95% CI	*p* value
	1.483	1.184–1.858	< 0.001
**Hospital stay (days)**	1.218	1.019–1.456	**0.021**
**Pre-op BUN (mg/dL)**	1.135	1.012–1.274	**0.034**
**Pre-op NLR**	1.341	1.091–1.648	**0.004**
**Pre-op PLR**	1.127	1.018–1.349	**0.029**
**Log-transformed preoperative SII**	1.261	1.052–1.512	**0.015**
**Post-op 7. Day Creatinine (mg/dL)**	4.755	2.043–11.071	**< 0.001**
**Post-op 7. Day NLR**	1.219	1.060–1.402	**0.006**
**Log-transformed Post-op Day 7 SII**	1.385	1.008–1.904	**0.042**

Model explanatory power: Nagelkerke R² =0.671 Adjusted OR: Adjusted Odds Ratio; CI: Confidence Interval; ICU: Intensive Care Unit; BUN: Blood Urea Nitrogen; NLR: Neutrophil-to-Lymphocyte Ratio; logSII7: Log-transformed Postoperative 7th Day Systemic Immune-Inflammation Index. Statistically significant p values are shown in bold

## Discussion

In our study, the decisive role of inflammatory parameters in predicting early clinical outcomes was clearly demonstrated in patients undergoing CABG, both in the preoperative and postoperative periods. Notably, in patients who experienced mortality, preoperative age, cardiopulmonary bypass and cross-clamp durations, as well as renal function tests (BUN and creatinine), were found to be significantly elevated. Additionally, increased preoperative NLR, PLR, and SII, together with a marked reduction in lymphocyte count, were observed. On the seventh postoperative day, patients with mortality exhibited significantly lower hemoglobin and platelet levels, whereas parameters such as neutrophil count, BUN, creatinine, sodium, and AST were significantly increased. In this period, inflammatory indices including NLR, PLR, and SII were markedly elevated, and lymphocyte count was further decreased. Importantly, postoperative day seven NLR and SII values showed the highest accuracy in predicting mortality. Moreover, the positive correlation between preoperative inflammatory parameters and extubation time, as well as length of hospital and intensive care stay, highlights the potential prognostic value of these markers in anticipating postoperative clinical course. In conclusion, both preoperative and postoperative inflammatory responses appear to significantly influence early prognosis, serving as meaningful predictors—especially for serious complications such as mortality and atrial fibrillation. These findings suggest that close monitoring of inflammatory markers and their incorporation into risk stratification may provide substantial benefits for patient management following coronary artery bypass surgery.

Beyond statistical significance, the clinical utility of inflammatory markers lies in their potential role in early risk stratification. In this study, postoperative day 7 NLR and SII demonstrated the highest predictive accuracy for early mortality, suggesting that patients exceeding these thresholds may represent a subgroup at increased risk for adverse postoperative outcomes. In routine clinical practice, such patients may benefit from closer hemodynamic monitoring, intensified surveillance for complications, and more individualized postoperative management. Importantly, these markers are derived from routinely available laboratory tests and can be readily integrated into daily clinical decision-making without additional cost or procedural burden.

From a pathophysiological perspective, elevated inflammatory indices reflect an exaggerated systemic inflammatory and immune response triggered by cardiopulmonary bypass and myocardial ischemia–reperfusion during CABG surgery. Cardiopulmonary bypass can provoke a systemic inflammatory reaction through innate immune activation, endothelial dysfunction, cytokine release, and oxidative stress, which may contribute to postoperative organ dysfunction and adverse outcomes [9]. Microvascular and endothelial injury after cardiac surgery is a key downstream consequence of this inflammatory response and may impair tissue perfusion, thereby linking heightened inflammatory burden to prolonged recovery and complications [10]. An increased neutrophil-to-lymphocyte ratio indicates a shift toward neutrophil-mediated innate inflammation accompanied by relative lymphocyte suppression, and perioperative NLR has been associated with worse outcomes after cardiac surgery in systematic evidence [11]. In parallel, platelet activation and platelet–leukocyte interactions have been described during cardiac surgery and are consistent with a thromboinflammatory milieu that can amplify microvascular injury and postoperative morbidity [12]. At the cellular level, activated neutrophils contribute to perioperative tissue injury through excessive production of reactive oxygen species, release of proteolytic enzymes, and formation of neutrophil extracellular traps, thereby amplifying myocardial, renal, and pulmonary damage. Concurrent lymphocyte apoptosis and stress-related immunosuppression impair adaptive immune regulation and are associated with adverse postoperative outcomes. In parallel, endothelial activation and cytokine-mediated capillary leakage promote microvascular dysfunction and organ hypoperfusion. In this context, the systemic immune-inflammation index may represent an integrated surrogate of immune dysregulation and thromboinflammatory burden rather than a simple numerical index. Collectively, these mechanisms provide a biologically plausible framework for the observed association between elevated NLR/PLR/SII and early complications, including mortality, atrial fibrillation, and prolonged postoperative recovery after CABG surgery.

In the study by Özer et al. (2018), the association between preoperative NLR and clinical outcomes was examined in patients undergoing coronary artery bypass surgery, revealing that those with higher preoperative NLR had significantly longer stays in both the intensive care unit and the hospital [13]. Additionally, a positive correlation was observed between preoperative NLR and the duration of both hospital and ICU stay. Similarly, our study found that, in addition to NLR, other inflammatory markers such as PLR and SII were also strongly associated with early clinical outcomes and mortality. Notably, elevated preoperative NLR and decreased lymphocyte count, as well as increased NLR and SII in the early postoperative period, stood out as significant predictors of mortality risk. Both studies underscore the substantial impact of preoperative inflammatory burden on postoperative recovery and prognosis. However, by also evaluating PLR and SII, our study provided a more comprehensive analysis of the inflammatory response and highlighted the predictive value of these parameters for major complications, including postoperative mortality and atrial fibrillation.

Tzikos et al. reported that postoperative abnormal PLR was associated with an increased risk of early mortality in patients undergoing cardiac surgery [14]. Nevertheless, no previous studies have specifically evaluated the impact of preoperative PLR on long-term mortality in patients who underwent CABG or OPCAB. Separately, Lee et al. assessed changes in RDW-SD following isolated CABG and demonstrated that alterations in RDW-SD independently predicted early postoperative adverse events [15]. Joshi et al. further expanded the understanding of RDW-SD by examining its prognostic value for in-hospital mortality in patients who did not undergo OPCAB, showing that abnormal RDW-SD was an independent predictor of this outcome [16]. Bujak et al. delved into the underlying mechanisms of abnormal RDW-SD in coronary artery disease, providing a molecular explanation for its adverse effects [17]. Additionally, Gurbuz et al. investigated the association between elevated RDW-SD and long-term cardiovascular event rates in CABG patients, finding a strong correlation between higher RDW-SD and increased incidence of these events [18]. In the study by Greberski et al. (2024), it was demonstrated that blood biomarkers such as preoperative NLR, PLR, and lymphocyte count were significant predictors of both early and late mortality in patients undergoing coronary artery bypass surgery [19]. The study emphasized that NLR and PLR values outside the normal range were associated with higher 30-day, 1-year, and 5-year mortality rates, and that especially elevated PLR was among the strongest predictors for both short- and long-term mortality. Similarly, in our study, increased preoperative NLR, PLR, and SII values and decreased lymphocyte counts were found to be significantly associated with both early mortality and adverse clinical outcomes such as prolonged hospital and ICU stay. Furthermore, while Greberski et al. also highlighted the importance of close monitoring of inflammatory markers in the postoperative period for prognostic assessment, our findings specifically identified NLR and SII on postoperative day seven as having the highest accuracy for predicting mortality.

In the study by Güngör et al. (2017), the relationship between postoperative atrial fibrillation and preoperative PLR was investigated, demonstrating that elevated PLR was an independent risk factor for the development of atrial fibrillation after coronary artery bypass surgery [20]. Both age and PLR were identified as independent predictors in multivariate analysis, and preoperative PLR was shown to be a significant indicator for postoperative atrial fibrillation. Consistently, our study found that, in addition to PLR, other inflammatory markers such as NLR and SII were also significantly associated with early postoperative complications, particularly atrial fibrillation, as well as with overall clinical outcomes. Notably, patients who developed atrial fibrillation exhibited markedly higher preoperative and postoperative inflammatory markers, and experienced a significantly prolonged ICU stay. Both studies suggest that assessment of preoperative inflammatory biomarkers may be valuable in predicting postoperative arrhythmias and complications. Furthermore, the inclusion of NLR and SII alongside PLR in our analysis allowed for a more comprehensive evaluation of the different aspects of the inflammatory response and its impact on clinical outcomes.

A variety of inflammatory biomarkers, such as white blood cell (WBC) count, different leukocyte populations, platelet levels, C-reactive protein (CRP), neutrophil-to-lymphocyte ratio (NLR), and platelet-to-lymphocyte ratio (PLR), have been identified as significant prognostic indicators in numerous cardiovascular conditions [21]. Distinct subtypes of WBCs—including neutrophils, lymphocytes, monocytes, and eosinophils—fulfill specific functions in the mechanisms of inflammation, immune defense, and tissue repair [22]. These immunological elements are central to the pathogenesis of organ injury, and their involvement is particularly relevant in the context of renal damage [23]. In the study by Parlar and Şaşkın (2018), it was shown that both preoperative and postoperative NLR and PLR values measured in patients undergoing isolated coronary artery bypass surgery were significantly associated with the development of early postoperative acute kidney injury (AKI) [24]. Multivariate analyses identified elevated NLR and PLR in both the preoperative and early postoperative periods as independent risk factors for AKI. Similarly, our study demonstrated that increased preoperative NLR, PLR, and SII were significantly related to early complications and mortality. Furthermore, both studies highlighted the importance of the preoperative inflammatory response as a biomarker for predicting clinical course; notably, the ease of obtaining parameters such as NLR and PLR from a routine hemogram provides a practical advantage in clinical practice. While the study by Parlar and Şaşkın emphasized the predictive value of NLR and PLR for early postoperative renal dysfunction, our research also established the association of these parameters with major clinical outcomes, including mortality and atrial fibrillation.

In addition to systemic inflammatory markers, biomarkers of myocardial damage have an established role in perioperative risk assessment in cardiac and cardiac surgical interventions. Cardiac troponin T is widely used as a sensitive indicator of myocardial injury and has been shown to provide prognostic information in patients undergoing cardiac surgery. Recent evidence suggests that perioperative troponin T measurements may help identify patients at increased risk of adverse outcomes and support clinical decision-making in the planning and early postoperative management of cardiac procedures [25]. Although troponin T was not included in the present analysis due to the retrospective design and lack of standardized measurements, its complementary role alongside inflammatory indices should be acknowledged.

This study has several limitations. First, as a single-center and retrospective study, the findings are subject to limitations in generalizability due to center-specific practice variations and the relatively limited patient population. In addition, center-specific factors such as surgical techniques, cardiopulmonary bypass strategies, anesthetic management, and perioperative intensive care protocols may have influenced postoperative inflammatory responses and clinical outcomes, thereby limiting the external validity of the findings. Although inclusion and exclusion criteria were applied meticulously, it was not possible to completely eliminate comorbidities or subclinical inflammatory processes. Additionally, although the timing of preoperative and postoperative laboratory parameter measurements was based on standardized protocols, minor differences in sampling times in clinical practice may have affected the results of inflammatory markers. Due to the retrospective design, serum troponin T levels were not available in a standardized manner for all patients and therefore were not included in the analysis. Due to the retrospective nature of the study, potential perioperative confounders such as blood transfusions, perioperative medication use (including inotropes, vasopressors, and anti-inflammatory agents), and postoperative infectious complications could not be systematically adjusted for, which may have influenced inflammatory markers and clinical outcomes. The generalizability of the findings is also limited by the relatively small patient cohort and the lack of validation in multicenter, prospective studies. Finally, only early-term outcomes were analyzed, and data regarding long-term mortality and morbidity were not included in this study.

This study demonstrated that preoperative and postoperative inflammatory parameters (NLR, PLR, and SII) are significantly associated with early clinical outcomes in patients undergoing coronary artery bypass graft surgery. Notably, elevated preoperative NLR, PLR, and SII values, as well as increases observed on postoperative day seven, emerged as independent risk factors for the prediction of mortality and major complications. Our findings suggest that inflammatory biomarkers should be incorporated into postoperative patient management and risk stratification after cardiac surgery. However, further validation of these results with larger, multicenter, and prospective studies is warranted.

## Data Availability

The data presented in this study are available on request from the corresponding author. The data are not publicly available due to ethical and privacy restrictions, as they contain sensitive information from child participants.
